# Influence of Time-Series Normalization, Number of Nodes, Connectivity and Graph Measure Selection on Seizure-Onset Zone Localization from Intracranial EEG

**DOI:** 10.1007/s10548-018-0646-7

**Published:** 2018-04-26

**Authors:** Pieter van Mierlo, Octavian Lie, Willeke Staljanssens, Ana Coito, Serge Vulliémoz

**Affiliations:** 1 Medical Image and Signal Processing Group, Ghent University, Ghent, Belgium; 2 Functional Brain Mapping Lab, University of Geneva, Geneva, Switzerland; 3 Department of Neurology, University of Texas Health Science Center at San Antonio, San Antonio, TX USA; 4 EEG and Epilepsy Unit, University Hospital of Geneva, Geneva, Switzerland

**Keywords:** Multivariate directed functional connectivity, Time-series normalization, Number of connectivity nodes, Epilepsy, Intracranial EEG, Granger causality

## Abstract

We investigated the influence of processing steps in the estimation of multivariate directed functional connectivity during seizures recorded with intracranial EEG (iEEG) on seizure-onset zone (SOZ) localization. We studied the effect of (i) the number of nodes, (ii) time-series normalization, (iii) the choice of multivariate time-varying connectivity measure: Adaptive Directed Transfer Function (ADTF) or Adaptive Partial Directed Coherence (APDC) and (iv) graph theory measure: outdegree or shortest path length. First, simulations were performed to quantify the influence of the various processing steps on the accuracy to localize the SOZ. Afterwards, the SOZ was estimated from a 113-electrodes iEEG seizure recording and compared with the resection that rendered the patient seizure-free. The simulations revealed that ADTF is preferred over APDC to localize the SOZ from ictal iEEG recordings. Normalizing the time series before analysis resulted in an increase of 25–35% of correctly localized SOZ, while adding more nodes to the connectivity analysis led to a moderate decrease of 10%, when comparing 128 with 32 input nodes. The real-seizure connectivity estimates localized the SOZ inside the resection area using the ADTF coupled to outdegree or shortest path length. Our study showed that normalizing the time-series is an important pre-processing step, while adding nodes to the analysis did only marginally affect the SOZ localization. The study shows that directed multivariate Granger-based connectivity analysis is feasible with many input nodes (> 100) and that normalization of the time-series before connectivity analysis is preferred.

**Electronic supplementary material:**

The online version of this article (10.1007/s10548-018-0646-7) contains supplementary material, which is available to authorized users.

## Introduction

In approximately 15–25% of the epilepsy patients in the presurgical evaluation, intracranial EEG (iEEG) monitoring is necessary to obtain additional localization information about the seizure-onset zone (SOZ) and eloquent cortex (Carrette et al. 2010). Intracranial EEG is recorded with stereo-EEG or depth electrodes inserted in the brain’s parenchyma, or with strip and grids placed on the top of the cortex. Because of the limited spatial sampling of iEEG, a clear hypothesis about the EZ must be available prior to electrode implantation. In clinical practice, the identification of the SOZ from iEEG is done visually by the epileptologist. This is a time consuming, labor intensive task that requires much expertise and suffers from interpreter dependency.

Functional brain connectivity is defined as the study of temporal correlations between spatially distinct neurophysiological events (Friston et al. 1993). Functional brain connectivity measures have been shown to localize the SOZ from iEEG recordings in an objective manner (van Mierlo et al. 2014). Multivariate connectivity measures based on the concept of Granger causality (Granger 1969), have been particularly successful in the context of SOZ localization. Granger causal modeling estimates whether a time series is useful to predict another. Accordingly, a signal x_1_ Granger-causes another signal x_2_, if the inclusion of the past values of x_1_ help to predict x_2_ beyond the information present in the past values of x_2_. Granger-causality measures use autoregressive (AR) models to parametrize the signal with a set of AR coefficients encoding the linear contribution of its recent past.

The two most commonly Granger-based measures used to localize the SOZ are the Directed Transfer Function (DTF) (Kaminski and Blinowska 1991) and the Partial Directed Coherence (PDC) (Baccalá and Sameshima 2001). They are multivariate estimators of the network connections between the EEG signals in the frequency domain, and thus, they require multivariate autoregressive (MVAR) models to estimate the present of each signal as a linear contribution of the past values of all signals. In 1994 and 1998, Franaszczuk et al. proposed that the SOZ can be localized in temporal lobe epilepsy patients based on visual interpretation of the propagation patterns derived from DTF analysis of ictal iEEG recordings (Franaszczuk and Bergey 1998; Franaszczuk et al. 1994). More recently, It has been shown that nodes with high outflow based on DTF calculations were highly correlated with the clinically identified foci in pediatric patients with neocortical epilepsy (Wilke et al. 2010a) and with Lennox-Gastaut syndrome (Jung et al. 2011). Furthermore, it has been shown that EZ estimation using DTF analysis mapped better the surgical resection area in patients with successful surgical outcome than those with unsuccessful outcome (Kim et al. 2010). Using the PDC, Baccalá et al. (2004) showed that correct epileptogenic focus localization was obtained by analyzing strongly connected subgraphs of scalp EEG and that the EZ can be localized from stereo-EEG in patients with epilepsy secondary to type II focal cortical dysplasia (Varotto et al. 2012).

All the above methods assumed stationary EEG signals in the analyzed window and therefore did not track the evolution of the connectivity pattern over time. Furthermore, time-varying methods allow modeling non-stationary signals such as the onset of a seizure. Two methods have been proposed for multivariate time-varying connectivity analysis: the short time DTF (SDTF) (Ding et al. 2000), where the DTF is calculated in a short time sliding window, and the adaptive (time-varying) DTF (ADTF) or PDC (APDC) (Astolfi et al. 2008; Wilke et al. 2008) based on Kalman-filter AR models. Mullen et al. (2011) used the first approach to analyze the connectivity between ICA components of ictal iEEG epochs, showing a shift in connectivity during the seizure. Wilke et al. (2010b) used the second approach to investigate the ability of the time-varying ADTF coupled to graph analysis measures to identify critical network nodes during the interictal states and compared this with the critical nodes identified with the DTF and graph analysis during ictal and resting interictal periods. They found that one graph measure, the betweenness centrality, correlated with the resected area in patients who were rendered seizure-free after surgery for all three investigated periods: ictal, resting interictal and interictal spikes. Furthermore, the gamma band was identified as the most important band to be studied because the betweenness centrality in this band correlated most with the resected area. In a previous study, we applied the ADTF coupled to outdegree to estimate the SOZ based on connectivity pattern changes/propagation in 8 patients rendered seizure-free by surgery, and found correspondence between these estimates, the visual iEEG analysis by the epileptologist, and the resected regions (van Mierlo et al. 2013). Furthermore, the obtained time-varying connectivity patterns were consistent in each patient over multiple seizures. For a more extensive overview of studies that localized the epileptogenic focus using functional connectivity we refer the readers to (van Mierlo et al. 2014).

Some important limitations apply to these previous studies. First, because of computational limitations, all previous studies using adaptive AR approaches have been applied to a limited number of iEEG channels/connectivity nodes (< 50–60), often selected based on visible involvement in the course of a seizure. Second, since the mathematical formulation of most AR models used has assumed white noise stationary processes with zero mean, normalization of iEEG time series by z-scoring has been suggested in several studies (Blinowska 2011; Kaminski and Blinowska 2014), but is not always done. The behavior of connectivity estimations using no or other normalization approaches has not been tested so far. Third, a comparison of the performance of several APDC and ADTF measures and graph theory measures used in the literature to localize the SOZ has not been carried out yet.

In this study, we assessed whether localizing the SOZ from a high number of iEEG channels is feasible. We investigated the influence of incorporating more nodes in the functional connectivity analysis. Furthermore, we studied how if we should normalize the time-series and which functional connectivity measure coupled to which graph measures are optimal to localize the SOZ. First, we performed simulations to quantify the performance of the different connectivity analyses. Second, we mapped the SOZ recorded in a high number of iEEG channels in an epilepsy patient and compared the obtained SOZ localization to the resection that rendered the patient seizure-free.

## Methods

In this section, we describe how we simulated seizures recorded with intracranial EEG electrodes. Later, we introduce the pipeline used to calculate the time-varying connectivity pattern and how we can localize the SOZ using graph theory measures. Afterwards, we explain how we have quantified the performance of the different connectivity measures coupled with the different graph measures. Finally, we describe how we have analyzed a 113-channel seizure recorded in a patient.

### Simulations

**Fig. 1:**
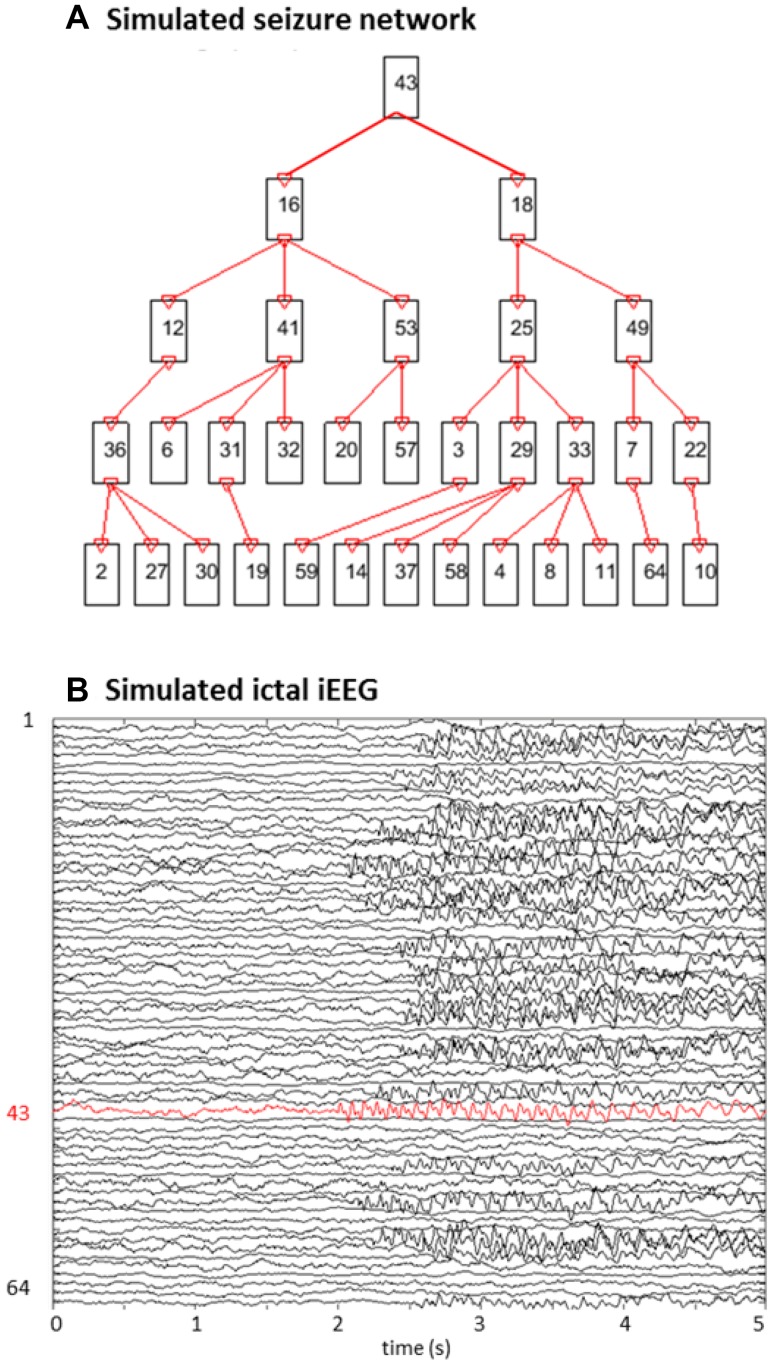
Example of simulated 64-channel iEEG during a seizure with SNR equal to 5 dB. In **a** the connectivity scheme is shown, **b** depicts the generated iEEG. Node 43 is the simulated SOZ. The seizure first spread to node 16 and 18 and later to nodes 12, 41, 53 from node 16 and to node 25 and 49 from node 18. Afterwards the seizure spreads as indicated in **a**

We simulated a seizure as recorded with 128 channels iEEG. The simulated iEEG is 5 s long, 2 s baseline followed by 3 s seizure. After the baseline duration, the seizure starts at a randomly chosen channel, the SOZ channel: *chan*_*SOZ*_. The seizure spreads to 31 other randomly chosen channels with the following parameters: maximal spreading from one channel to three other channels, random onset delay, i.e. the time a node gets involved in the seizure, between 1 and 250 ms and sample delay, i.e. the number of samples the signal of the sending node is delayed with respect to the receiving node, randomly chosen between 1 and 5 samples. In total, 32 channels will show seizure activity. Figure 1 shows a possible seizure network and the ictal iEEG signals. The baseline activity was modeled by 1/f noise and the seizure activity as a time-varying sinusoid with frequency equal to 12 Hz at the start of the seizure (at 0 s) and equal to 8 Hz at the seizure end (at 3 s) plus its first harmonic (van Mierlo et al. 2011). This way we want to mimic the rhythmic phase of the seizure, i.e. the period with periodic ictal spiking. The simulations were inspired by the oscillatory patterns seen in a real seizure originating from the temporal lobe (van Mierlo et al. 2011). The signal-to-noise ratio (SNR) of the seizure activity compared to the baseline activity was set to − 5, 0, 5 and 10 dB. The amplitude of the different channels was chosen randomly between 25 and 100 mV to mimic amplitude changes observed in the iEEG. An overview of the parameters used to simulate the iEEG seizures is shown in Table 1.

**Table 1:** Simulation parameters

Sampling frequency	200 Hz
Number of channels	128
Number of ictal channels	32
Baseline duration	3 s
Seizure duration	5 s
Sample delay	1–5
Onset delay	1–250 ms
Max number of propagated channels	3
SNR	− 5, 0, 5, 10 dB
Seizure signal frequency	12–8 Hz

### From iEEG to SOZ Localization

The complete pipeline to localize the SOZ from the iEEG recordings is introduced below. It comprises three subparts: pre-processing, connectivity calculation and graph analysis. Below we give a detailed overview of all subparts.

#### Pre-processing

The pre-processing consisted of two steps: a channel selection step and a time series normalization step. In the channel selection step, 32, 64, 96 or all 128 channels were chosen to investigate the effect of adding more nodes to the functional connectivity analysis. The 32 ictal channels were included in all channel selections. In the normalization step, the different options were: no normalization, z-scoring of the complete 5 s time series, sliding window z-scoring with a window length equal to 1 s and baseline z-scoring, where only the first 2 s are used to calculate the mean and standard deviation to perform z-scoring. Due to the preprocessing, each simulation resulted in 16 different datasets that were processed further as indicated below.

#### Multivariate Time-Varying Connectivity Analysis

In this section, we introduce the different time-varying functional connectivity measures that are used to estimate the dynamic information flow between the considered signals. All measures used in this study are based on the concept of Granger causality. A common method to study Granger causality is to use autoregressive models in which the influence of the past of the signals on the present is estimated. The time-varying bivariate autoregressive (TVAR) model of signal x_1_ and x_2_ can be described as:

1 $${x_1}\left( t \right)=\mathop \sum \limits_{{m=1}}^{p} {a_{11,m}}\left( t \right){x_1}\left( {t - m} \right)+\mathop \sum \limits_{{m=1}}^{p} {a_{12,m}}\left( t \right){x_2}\left( {t - m} \right)+~{e_1}\left( t \right)~~$$

2 $${x_2}\left( t \right)=\mathop \sum \limits_{{m=1}}^{p} {a_{21,m}}\left( t \right){x_1}\left( {t - m} \right)+\mathop \sum \limits_{{m=1}}^{p} {a_{22,m}}\left( t \right){x_2}\left( {t - m} \right)+~{e_2}\left( t \right)$$

where a_12_, a_12_, a_21_ and a_22_ are the TVAR coefficients and e_1_ and e_2_ the residuals of signals x_1_ and x_2_.

The bivariate case can be extended to the multivariate case with *K* channels as follows:

3 $${\varvec{X}}\left( t \right)=\mathop \sum \limits_{{m=1}}^{p} {{\varvec{A}}_m}\left( t \right){\varvec{X}}(t - m)+{\varvec{E}}(t)$$

where **X**(t) is the iEEG matrix, **A**_m_(t) are the model coefficients matrix for delay *m* and **E**(t) is the residual matrix. The coefficients of the TVAR model were estimated using a Kalman filter with update coefficient equal to 10^−3^ and Kalman smoothing term equal to 100 (van Mierlo et al. 2011). To investigate the information flow between the nodes in the spectral domain, we apply the Fourier transform to the coefficient matrices at each time point *t* as follows:

4 $${\varvec{A}}\left( {f,t} \right)={{\varvec{I}}_{\varvec{K}}} - \mathop \sum \limits_{{m=1}}^{p} {{\varvec{A}}_m}\left( t \right){\text{exp}}\left( { - i2\pi \frac{f}{{{f_s}}}m} \right)$$

where **I**_**K**_ is the *K* times *K* identity matrix. From the Fourier transform of the coefficient matrices **A**(f,t) the transfer matrix **H**(f,t) is calculated:

5 $${\varvec{H}}\left( {f,t} \right)={\varvec{A}}{(f,t)^{ - 1}}$$

The element **A**_**ij**_(f,t) and **H**_**ij**_(f,t) depict the information flow from node *j* to node *i* at frequency *f* and time *t*. In **H**(f,t) the cascade flow is modeled, while in **A**(f,t) only the direct flows are modeled. This means that if there is a connection from node 1 to node 2 and from node 2 to node 3 at frequency *f* and time *t*, elements **A**_**21**_(f,t) and **A**_**32**_(f,t) will have a high value, while **H**_**21**_(f,t) and **H**_**31**_(f,t) will be high.

Different normalizations of the **A**(f,t) and **H**(f,t) matrices are performed to scale the connectivity values between 0 (no connection) and 1 (high connection). Because in the next step, we will examine the outflow of the nodes, we use the Adaptive Partial Directed Coherence (APDC) and the Adaptive Directed Transfer Function (ADTF) normalized with respect to the inflow at each time *t* and frequency *f* as suggested by (Coito et al. 2015; Plomp et al. 2015):

6 $$APD{C_{ij}}\left( {f,t} \right)=\frac{{{{\left| {{{\varvec{A}}_{ij}}(f,t)} \right|}^2}}}{{\mathop \sum \nolimits_{{k=1}}^{K} {{\left| {{{\varvec{A}}_{ik}}(f,t)} \right|}^2}}}$$

7 $$ADT{F_{ij}}\left( {f,t} \right)=\frac{{{{\left| {{{\varvec{H}}_{ij}}(f,t)} \right|}^2}}}{{\mathop \sum \nolimits_{{k=1}}^{K} {{\left| {{{\varvec{H}}_{ik}}(f,t)} \right|}^2}}}$$

The following normalization holds for both ADTF and APDC:

8 $$\mathop \sum \limits_{{j=1}}^{K} APD{C_{ij}}\left( {f,t} \right)=1$$

The ADTF and APDC can be integrated in the frequency band of interest to estimate the connections over time in the considered frequency band, which results in the integrated APDC and ADTF (iAPDC and iADTF) and the full-frequency APDC and ADTF (ffAPDC and ffADTF):

9 $$iAPD{C_{ij}}\left( t \right)=\frac{1}{{{f_2} - {f_1}}}\mathop \sum \limits_{{f={f_1}}}^{{{f_2}}} \frac{{{{\left| {{{\varvec{A}}_{ij}}\left( {f,t} \right)} \right|}^2}}}{{\mathop \sum \nolimits_{{k=1}}^{K} {{\left| {{{\varvec{A}}_{ik}}\left( {f,t} \right)} \right|}^2}}}$$

10 $$iADT{F_{ij}}\left( t \right)=\frac{1}{{{f_2} - {f_1}}}\mathop \sum \limits_{{f={f_1}}}^{{{f_2}}} \frac{{{{\left| {{{\varvec{H}}_{ij}}(f,t)} \right|}^2}}}{{\mathop \sum \nolimits_{{k=1}}^{K} {{\left| {{{\varvec{H}}_{ik}}(f,t)} \right|}^2}}}$$

11 $$ffAPD{C_{ij}}\left( t \right)=\mathop \sum \limits_{{f={f_1}}}^{{{f_2}}} \frac{{{{\left| {{{\varvec{A}}_{ij}}(f,t)} \right|}^2}}}{{\mathop \sum \nolimits_{{f'={f_1}}}^{{{f_2}}} \mathop \sum \nolimits_{{k=1}}^{K} {{\left| {{{\varvec{A}}_{ik}}(f',t)} \right|}^2}}}$$

12 $$ffADT{F_{ij}}\left( t \right)=\mathop \sum \limits_{{f={f_1}}}^{{{f_2}}} \frac{{{{\left| {{{\varvec{H}}_{ij}}(f,t)} \right|}^2}}}{{\mathop \sum \nolimits_{{f'={f_1}}}^{{{f_2}}} \mathop \sum \nolimits_{{k=1}}^{K} {{\left| {{{\varvec{H}}_{ik}}(f',t)} \right|}^2}}}$$

The iAPDC, iADTF, ffAPDC and ffADTF are normalized with respect to incoming information flow at each time point *t*. This means that the following normalization holds for all measures:

13 $$\mathop \sum \limits_{{j=1}}^{K} iAPD{C_{ij}}(t)=1$$

The ADTF measures are able to reveal cascade connections, while the APDC measures reveal the direct connections. The ADTF can be used to identify the origin of information flow and the APDC to identify the direct connections.

#### Graph Measures to Localize the SOZ

Two graph measures are calculated for each time-varying connectivity measure to localize the SOZ: the outdegree and the shortest path length. The outdegree calculates the sum of the outflow from one node to all the other nodes, while the shortest path length defines the shortest paths from one node to another.

The global outdegree was defined as the out-degree during the seizure for each node:

14 $${\omega _j}=\mathop \sum \limits_{{t={t_1}}}^{{{t_2}}} \mathop \sum \limits_{{k=1}}^{K} {C_{kj}}(t)$$

here, *t*_*1*_ is the seizure onset and *t*_*2*_ is the seizure end, *K* is the number of channels and C_kj_ is the connection from node *j* to node *k* calculated with the above described time-varying connectivity measures, meaning that C_kj_ is equal to iADTF_kj_, ffADTF_kj_, iAPDC_kj_ or ffAPDC_kj_. The node with maximal global outdegree is chosen as SOZ, because this node has maximum outflow during the seizure in the estimated time-varying network.

The second considered graph measure is the global shortest path length that is defined as the sum of shortest path lengths from one node to all other nodes:

15 $${\varphi _j}=\mathop \sum \limits_{{t={t_1}}}^{{{t_2}}} \mathop \sum \limits_{{k=1}}^{K} {\sigma _{kj}}(t)$$

where, σ_kj_ is the shortest path length from node j to node k in the graph where the edge weights between the nodes were set to 1/C_kj_. This means that when there is a high connection from j to k, the edge weight will be low and therefore the shortest path will be low. The node with the minimal global shortest path was identified as the SOZ, because it has the overall shortest path to all other nodes.

### Evaluation of the Simulations

We use two evaluation measures to evaluate the importance of the processing steps, namely the area under the curve (AUC) and the number of correctly localized SOZ.

#### AUC Analysis

For each simulated high dimensional intracranial EEG of a seizure, 256 time-varying connectivity matrices were calculated. One for each combination of the following factors:

- Channel selection: 32, 64, 96 or 128
- SNR: − 5, 0, 5 or 10 dB
- Normalization: none, z-score, sliding z-score or baseline z-score
- Connectivity measures: iADTF, ffADTF, iAPDC or ffAPDC

The order of the TVAR model to calculate the connectivity measures was set to 5, the update coefficient to 0.001 and the Kalman smoothing term to 100 (van Mierlo et al. 2011). The frequency band of interest was set to 3–30 Hz.

Each estimated time-varying connectivity matrix was compared to the true time-varying connectivity matrix of the simulation. This was done by calculating the true positives (TP), false positives (FP), true negatives (TN) and false negatives (FN) when comparing the thresholded estimated time-varying connectivity matrix with the ground truth. For the APDC measures the direct edges of the simulation were considered as ground truth, while for the ADTF the cascade connections were considered as ground truth. We let the threshold range from 0 to 1 in steps of 0.01 to calculate the TP, FP, TN and FN. Afterwards we computed the sensitivity and the precision, also known as the positive predictive value.

From the sensitivity and precision values we derived the AUC. Afterwards, we performed a univariate ANOVA with AUC as dependent variable and four factors: the SNR, connectivity measure, normalization and channel selection. Bonferroni correction was applied to correct for multiple comparisons.

#### SOZ Localization

Based on each time-varying connectivity measure we localized the SOZ based on the outdegree and shortest path. For the 100 simulations we assessed the percentage of correctly identified seizure onset zones. We compared the identified SOZ based on the outdegree and on the shortest path length with the simulated SOZ (*chan*_*SOZ*_).

### High Dimensional Intracranial Recordings in Patients

**Fig. 2:**
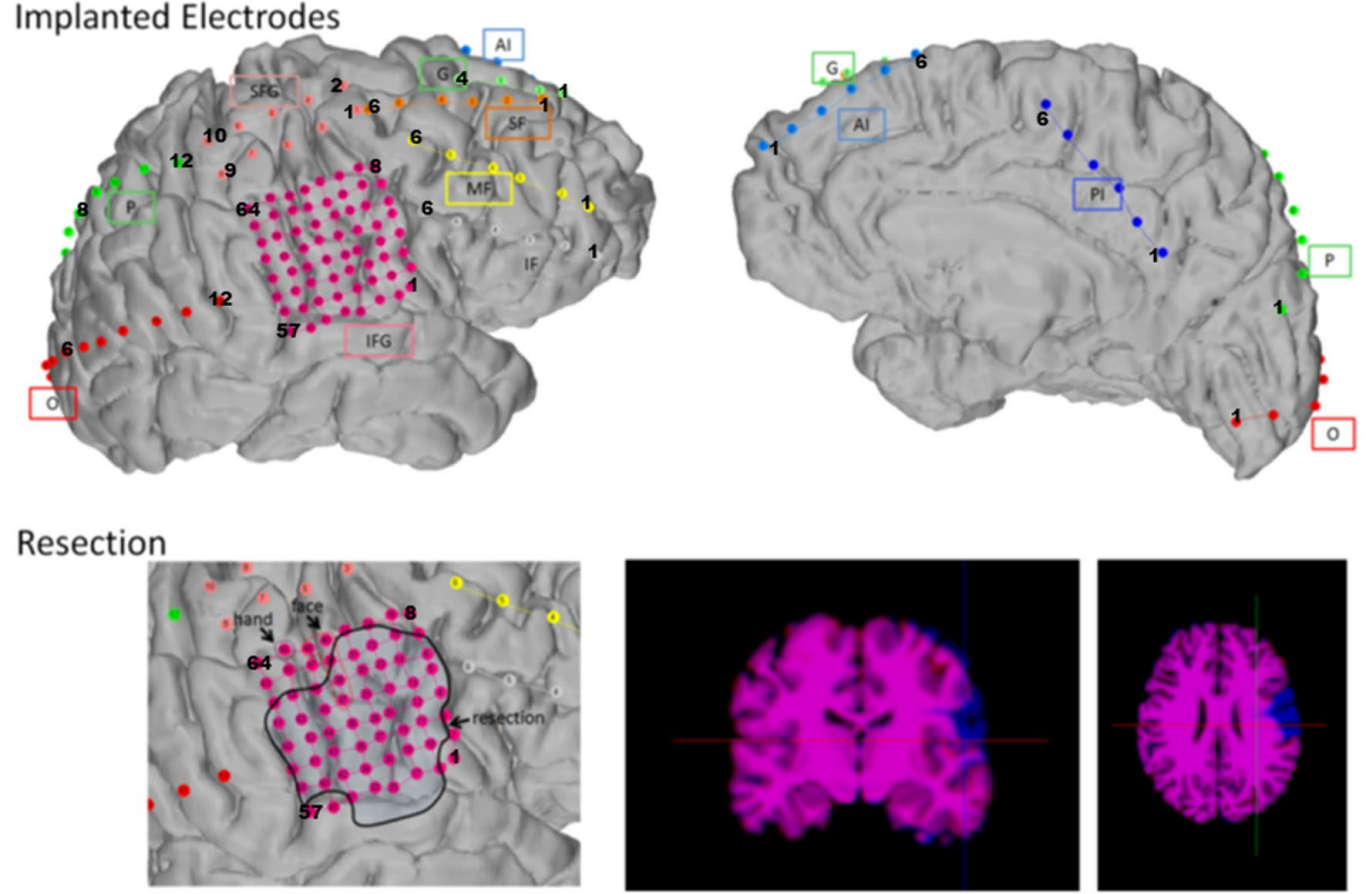
Implantation scheme of the 113 intracranial electrodes in the patient and the subsequent resection (black line on lower left panel and blue zone on lower right panel) that rendered the patient seizure-free

We investigated an ictal epoch recorded with 113 electrodes in the University of Texas Health Center at San Antonio. The patient had a MRI-positive focal cortical dysplasia type II b in the right perisylvian areas. The implantation scheme of the electrodes is shown in Fig. 2. An 8 × 8-contact inferior frontal grid (IFG, interelectrode distance: 5 mm) overlaid the lesion. An additional 2 × 5 superior frontal grid (interelectrode distance: 1 cm) and multiple strips were placed around IFG and interhemispherically. A 2-contact recording reference (G) was placed over the anterior mesial portion of the right superior frontal gyrus, remote from areas of high cortical irritability. The iEEG was sampled at 500 Hz. Visual analysis of the iEEG showed that the lesion was causing the epilepsy. Removal of the lesion rendered the patient seizure free and the patient has been seizure free since 3 years. The intracranial EEG signals of the analyzed seizure and their spectrogram can be found in the supplementary material.

### Evaluation of the Patient Data

We performed the proposed methods to localize the SOZ on the high dimensional iEEG epoch that consisted out of 5 s pre-ictal and 30 s ictal activity identified by visual analysis of the epileptologist (OL). We analyzed the full 113-channel iEEG and two subsets: one containing all 64 grid electrodes and one containing 25 electrodes closest to the visually identified SOZ. This was done to investigate the variation of SOZ localization using the proposed method when nodes are added to the connectivity analysis. We performed the four types of normalization and downsampled the time series to 250 Hz. Afterwards we calculated the iADTF, ffADTF, iAPDC, ffAPDC in combination with the outdegree and the shortest path length to localize the SOZ. The model order of the TVAR model was set to 8, update coefficient to 0.001 and kalman smoothing parameter to 100 (van Mierlo et al. 2011). The frequency band of interest was 2–30 Hz since the harmonic seizure frequency lied within this band. We compared the localized SOZ with the surgical resection that rendered the patient seizure free and investigated the influence of the normalization, the number of nodes and the used connectivity measures in combination with the graph measure.

## Results

### Simulations

#### AUC Analysis

**Fig. 3:**
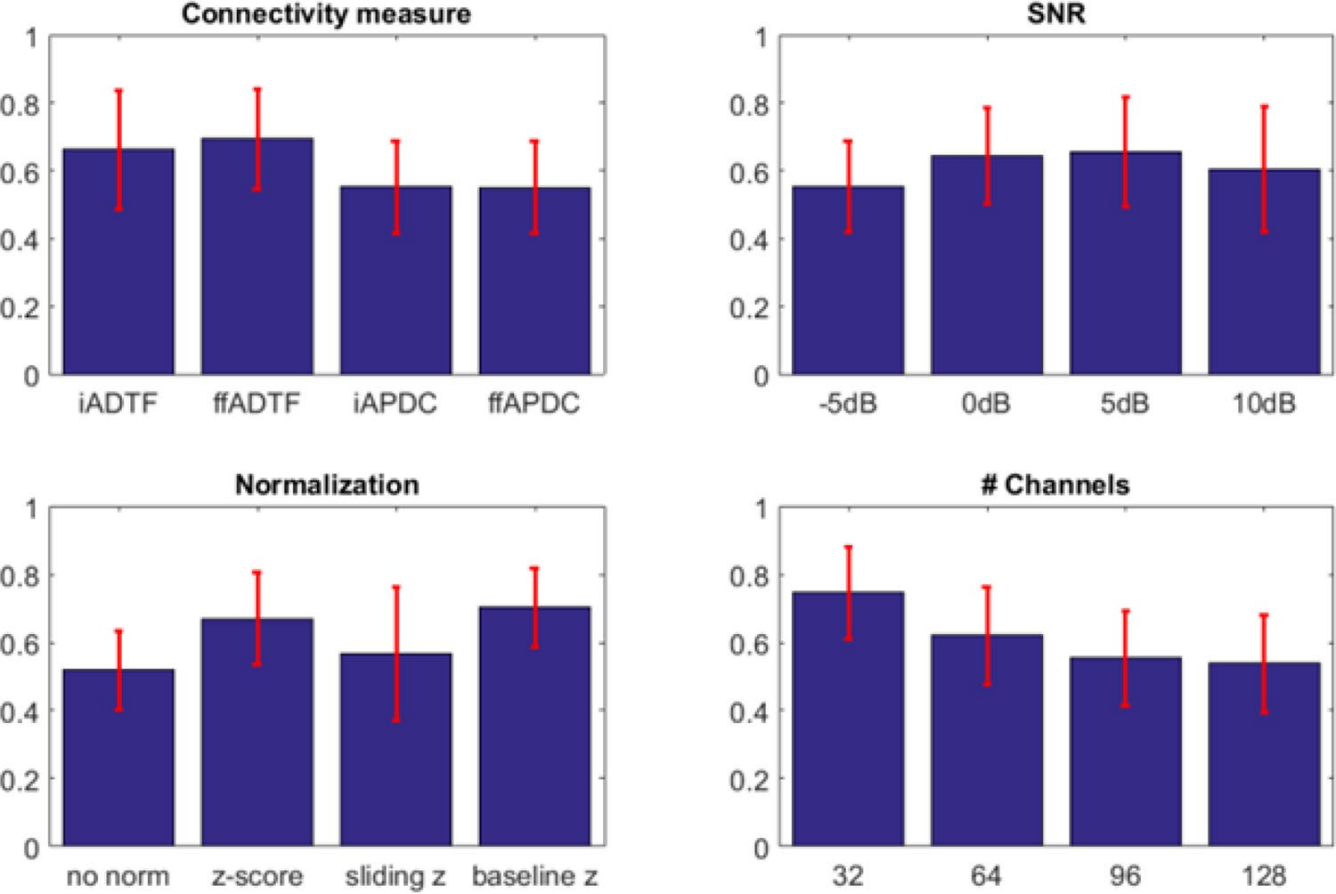
The AUC results of 100 seizure onset simulations. The blue bars indicate the mean value and the red error bars show the standard deviation. The left and right top panel shows the AUC with respect to the connectivity measures and the SNR, while the left and right bottom panel show the AUC with respect to normalization and number of channels, respectively

The statistical analysis of the AUC values revealed that the normalization, the channel selection, the SNR and the chosen connectivity measures all significantly influence the connectivity analysis. The results of the AUC analysis are shown in Fig. 3. The ADTF measures have a higher AUC than the APDC measures. The differences between integrated and full-frequency variants of the ADTF and APDC are less outspoken. We notice an increase in AUC when the SNR increases, with a small drop at 10 dB. This is because the difference between the baseline and seizure signal is high here resulting in non-stationarities that the TVAR model cannot easily model. When considering the normalization, we see that no normalization results in the worst AUC. Z-scoring the whole time series or the baseline provides the best results. The number of nodes influences the AUC. The more channels are used, the lower the AUC.

#### SOZ Localization

**Fig. 4:**
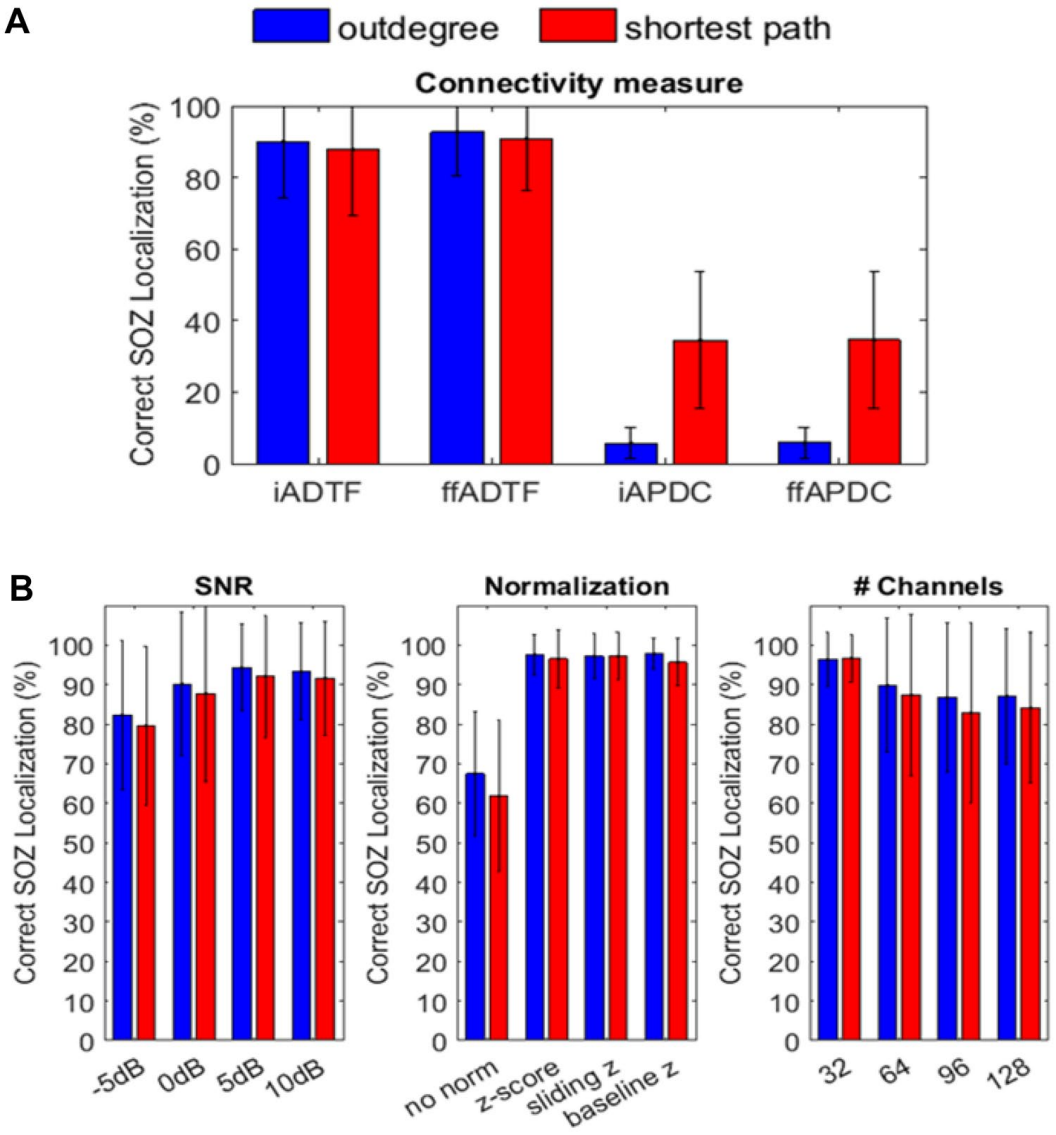
The number of correctly localized SOZ in the 100 simulations of a seizure onset. The bars indicate the mean value and the error bars represent the standard deviation. **a** shows the difference between the connectivity measures (ADTF and APDC) and the coupled graph measures (outdegree and shortest path). **b** shows the results for the integrated ADTF with respect to SNR, normalization and number of channels

The AUC tells us how well the time-varying connections are estimated, but more importantly, how well can we localize the SOZ from the time-varying connectivity matrix using the outdegree and shortest path length? The number of correctly localized SOZ is shown in Fig. 4.

In panel A the difference between the used connectivity measures coupled to the graph measures is shown. It can be noticed that the ADTF measures are preferred to localize the SOZ. The choice of the graph theory index (outdegree and shortest path) and of the connectivity measure (integrated or full-frequency) does not seem to play an important role for the ADTF. We achieve a mean of 90% correctly localized SOZ. For the APDC, the shortest path length is the preferred graph measure, achieving a mean of around 35% correct SOZ localizations.

In panel B, we zoom in on the results for the ADTF because these measures are preferred over the APDC to localize the SOZ. We only show the results of the iADTF, because the results of the ffADTF are highly similar. We notice that the normalization has the biggest influence on the results. No normalization results in 60–70% correctly localized SOZ, while with normalization (z-scoring, sliding window z-scoring or baseline z-scoring) we achieve a mean of approximately 95% correctly localized SOZ. This is an increase of 25–35% above what the analysis of un-normalized datasets achieves. We further notice that the number of correctly localized SOZ increases with the SNR and decreases around 10% with the number of nodes involved in the analysis as expected. The influence of the number of nodes is however much smaller than the normalization.

### Patient Recordings

We analyzed 12 datasets of the same seizure: four types of normalization and three groups of channels (25, 64 and 113). For each dataset we computed the iADTF, ffADTF, iAPDC and ffAPDC and then used the outdegree and shortest path length to localize the SOZ.

**Fig. 5:**
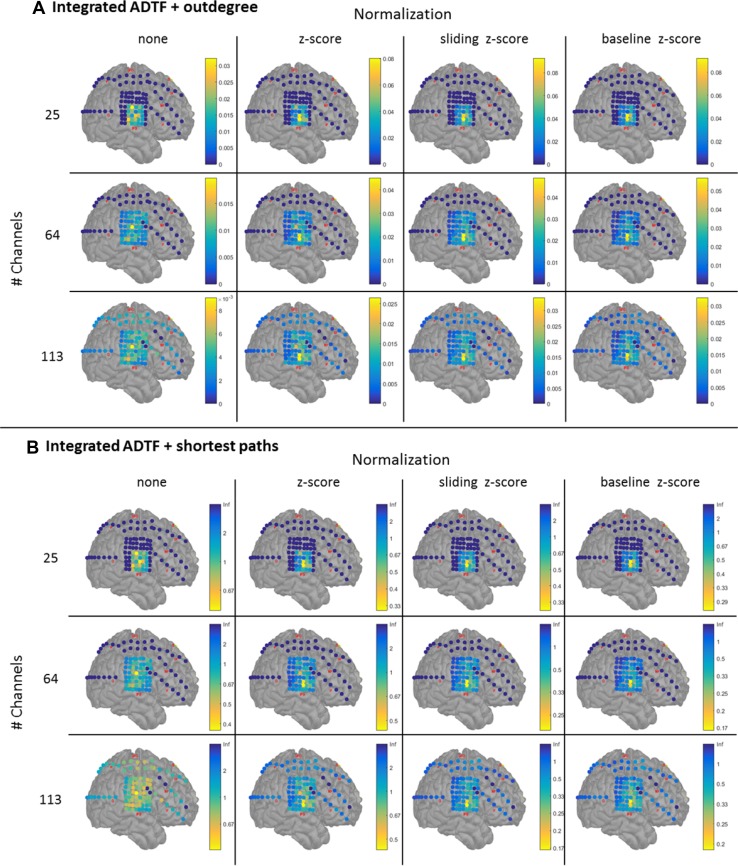
Seizure onset zone localization using the functional connectivity measure integrated ADTF coupled to outdegree (**a**) and shortest path (**b**) for different number of pre-selected channels (dark blue channels are not included) and different normalization of the time series. The results show that when normalization is performed the SOZ is focally localized in the resected volume and the pattern stays consistent when more channels are incorporated in the analysis. When the time series are not normalized a more widespread SOZ is found. Outdegree and shortest path lead to similar SOZ localization

The results for the iADTF coupled to the outdegree and shortest path are shown in Fig. 5. The results of the ffADTF are not shown but are similar. We notice that normalization has the biggest effect on the SOZ localization. All types of z-scoring led to the same results, namely IFG26-27 was identified as SOZ. The number of analyzed channels did not affect SOZ localization, meaning that the same SOZ was found when 25, 64 or 113 channels were considered. When no normalization was performed, a more diffuse SOZ localization pattern was found both for iADTF coupled either to the outdegree or to the shortest path. Here, the highest peaks were found for IFG37 and IFG42.

**Fig. 6:**
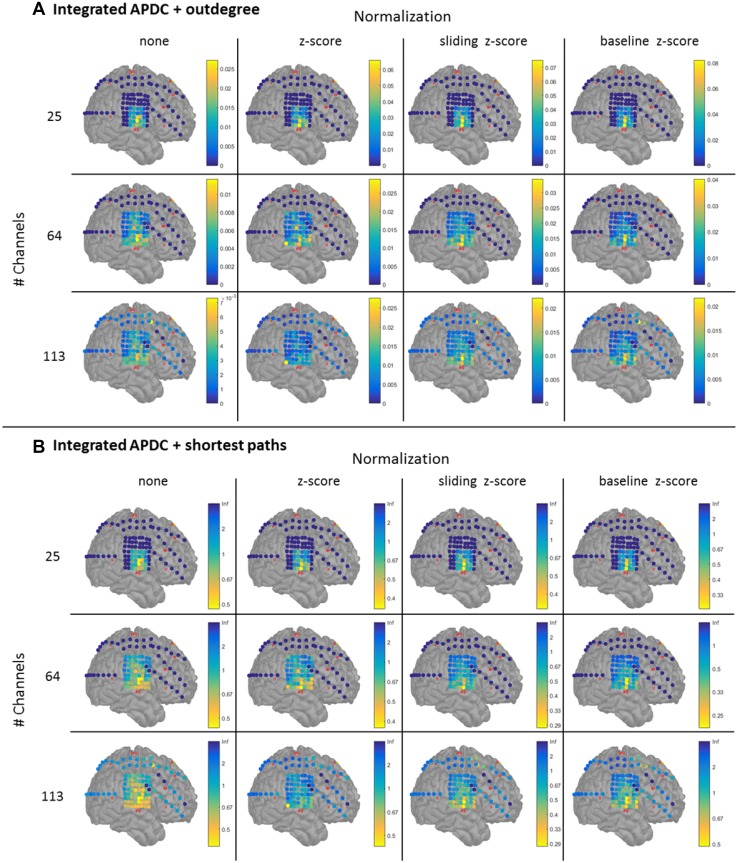
Seizure onset zone localization using the functional connectivity measure integrated APDC coupled to outdegree (**a**) and shortest path (**b**) for different number of pre-selected channels (dark blue channels are not included) and different normalization of the time series. The results show that when outdegree is used the SOZ is sometimes estimated outside the resection volume. Normalizing the time series using sliding z-scoring or baseline z-scoring in combination with shortest path depict the same SOZ as with the iADTF analysis (Fig. 5). Outdegree works when a limited number of channels are incorporated in the analysis, but fails when more channels are added. The SOZ pattern is not consistent when more channels are investigated

The results for the SOZ localization using the iAPDC are shown in Fig. 6. When more channels are added to the analysis, the iAPDC in conjunction with the outdegree or shortest path is not able to correctly identify the SOZ. When 64 or 113-channels are included in the analysis, electrode IFG57 or SF6 are found as SOZ depending on the normalization. Both electrodes do not correspond to the resection that rendered the patient seizure-free. When iAPDC is coupled to the shortest path length, its performance in localizing SOZ is more consistent when more channels are included in the analysis. In conclusion, SOZ localization obtained with ADTF analysis coupled to the outdegree or shortest path outperforms the APDC analysis.

## Discussion

Despite the use of a simple model of the seizure onset, our simulations provide insight into the steps to calculate directed functional connectivity. The normalization applied to the time series is the most important factor influencing the SOZ localization results. This is related to the amount of spurious connections estimated from low to high amplitude signals, because low amplitude signals more easily fit in large amplitude signals. Consider a bivariate TVAR model where x_1_ time-series has much higher amplitude than x_2_. Given the amplitude difference of the signals, coefficients a_12,m_(t) and a_21,m_(t) will have a much larger and smaller value, respectively, than the other coefficients in Eqs. 1 and 2. This results in a high connectivity from x_2_ to x_1_, which is actually spurious. Therefore, normalizing the time series before TVAR modeling is one way to solve the scaling issue and the resulting erroneous connections. Another way is by incorporating the covariance of the corresponding residuals in the formulas of the PDC and DTF, resulting in the so called generalized PDC and DTF (Baccala et al. 2007).

Beyond normalization, Florin et al. showed that filtering and decimation are important pre-processing steps (Florin et al. 2010). In principle, no filtering or filtering with a low model order is advised. Decimation by a factor greater than the minimum time shift between the time series may lead to wrong inferences. Florin et al. conclude that multivariate causality measures are very sensitive to data preprocessing, which we confirmed in our study by showing the sensitivity to time series normalization.

**Fig. 7:**
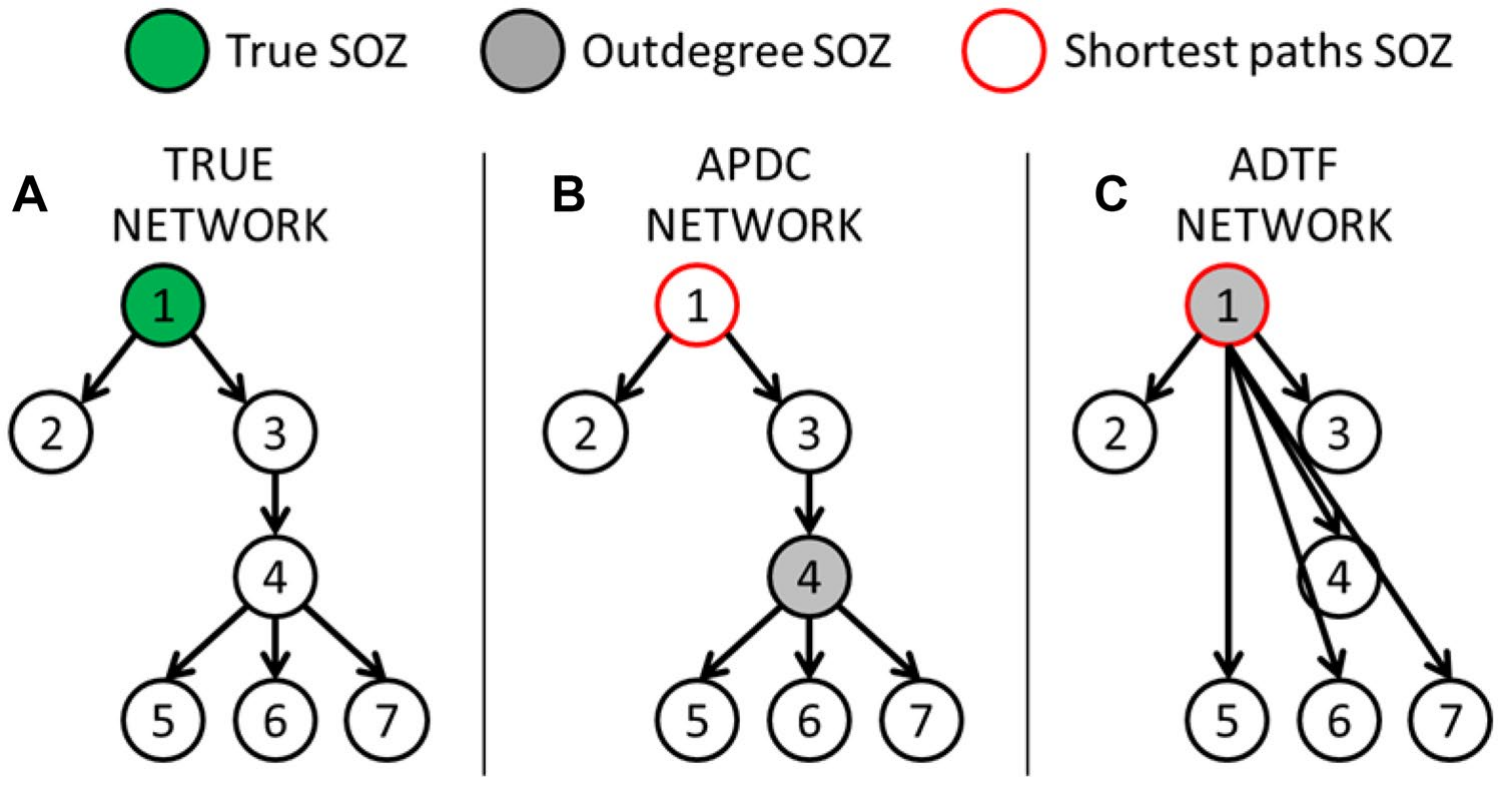
Illustrative example to indicate why outdegree does not work in combination with APDC to localize the SOZ, while it does work for ADTF. Shortest path analysis can correctly localize the SOZ for both ADTF and APDC in this example

The ADTF measures outperform the APDC measures to localize the SOZ. This is expected because ADTF models the indirect connections, and therefore, it indicates the origin of information flow, while the APDC models the direct connections. The outdegree and shortest path length performed equally well for the ADTF. For the APDC, the outdegree resulted in a low performance. This is because another node down the cascade can have a higher number of strong direct connections to other nodes, resulting in a higher out-degree. The global shortest path length estimates the distance between the node and all the other nodes and therefore, nodes from where the seizure originates will have smaller shortest path length since they are connected to all the nodes down the cascade. Consider the next illustrative example. We have a network of seven nodes that are connected as shown in Fig. 7a. The ADTF and APDC results are shown in Fig. 7b and c, respectively. Because node 4 has most outgoing connections in panel B, it will be depicted as SOZ when using outdegree. Calculating the shortest path to all other nodes can help to identify the SOZ for the APDC. For the ADTF the choice of graph measure is not important because it already models the indirect information flow as shown in Fig. 7c. Here, we show that both the outdegree and shortest path length can be used to localize the SOZ.

In the case of estimating the origin of information flow, which is desired to localize the SOZ or the irritative zone, ADTF measures are preferred over APDC. However, when one want to investigate the entire network or specific links between certain brain regions, the APDC is more appropriate. This means that both measures are valuable depending on the desired application.

To localize the irritative or seizure onset zone, a simple graph measure such as outdegree performs well (Coito et al. 2015, 2016b; van Mierlo et al. 2013). In these cases the use of outdegree suffices because we want to identify the source of the spike or seizure and the outdegree enables us to find the source where most information flow is coming from. To answer more complex research questions, such as ‘what are critical nodes in the epileptogenic network, which nodes behave abnormally?’ the use of more complex graph measures, such as shortest path and betweenness centrality, is beneficial as has been shown by (Wilke et al. 2010b). These and other graph measures allow investigating local and global properties of the network, enabling to answer more complex questions than what brain region is the driver of the recorded activity. The use of a graph measure also depends on the connectivity measure that is used to identify the connections. Investigating the outdegree when the edges are calculated using the (A)DTF or the (A)PDC gives a completely different picture of the brain, because (A)DTF models indirect connections and (A)PDC models only direct connections. Therefore, the choice of graph measure and connectivity measures depends on the research question one wants to answer and should be made carefully. Furthermore, it could be beneficial to study different graph measures simultaneously because they provide complementary characterizing information that allows a more profound analysis of the role of each node in the network (Amini et al. 2010).

The presented method localizes the channels most involved in the SOZ, but does not tell anything about the extent of the SOZ. In Fig. 5 it can be noticed that especially two electrodes have high out-degree. However, it is very unlikely that the patient would have become seizure free by the resection of this small area. The developed method indicates which brain region sends most out-going information during the seizure, but does not tell anything about the extent of the epileptogenic zone. Most likely, the SOZ as estimated by our method is comprised in the epileptogenic zone. For the method to be used in clinical practice it would be of great added value that not only the localization but also the extent of the SOZ can be derived from the connectivity graph. Further developments in this context, for example looking at locally connected subgraphs during the seizure onset to define the extent of the SOZ, could accelerate bringing these techniques to the clinical practice.

In clinical practice, the intracranial EEG is visually scanned for rapid discharges that are considered a characteristic pattern of the epileptogenic zone in focal epilepsy (Alarcon et al. 1995; Allen et al. 1992; Wendling et al. 2003). Wendling et al. showed that there is desynchronization between the different channels during this rapid discharge phase (Wendling et al. 2003). The rapid discharge phase is usually followed by ictal spiking during which the seizure spreads to many channels. In our study we focused on the ictal spiking period to extract the SOZ using functional connectivity, because we need the seizure to have spread to several channels to be able to use functional connectivity to localize were the seizure originated from. Another approach to localize the SOZ is the so-called ‘Epileptogenicity index’, where spectral and temporal features of the signals, namely the occurrence of fast activity and the temporal delay with regard to the seizure onset, are used to localize the epileptogenic zone. In a recent study, the link between preictal connectivity patterns and the epileptogenicity index was shown (Courtens et al. 2016). Based on stereo-EEG measurements of 24 seizures, correspondence was found between electrodes with high out-degree and total degree in the 15–40 Hz band and those with a high epileptogenicity index. Further studies comparing the rapid discharge phase and the functional connectivity patterns derived during different phases of the seizure would be of great interest.

In the simulations, signals are delayed from one channel to another to mimic propagation. At each channel 1/f noise is added that is also propagated to the next channel. This allows differentiation of the model where there is indirect transmission of information from node 1 to node 3 over node 2 (1–>2–>3) with the model using direct transmission from node 1 to node 2 and node 3 (1–>2 and 1–>3). In future studies more realistic simulations of a seizure, for example realistic models of coupled neuronal populations (Wendling et al. 2010) could be more informative of the behavior of the designed method in more realistic simulations. Nevertheless, the simple model used in our study is useful to highlight the methodological differences between the different connectivity measures coupled to the different graph measures.

During the simulation, we used the precision instead of specificity to quantify the performance, because there are many true negatives in the simulation. This means that the specificity has a value close to 1, because TN > > FP. A better measure to quantify the false positives in this case is the precision, because it does not depend on the large amount of TN (Saito and Rehmsmeier 2015).

The number of nodes has an influence on the calculated connectivity pattern, although this influence is rather small. For the patient data, we see that normalization affects the results more than adding extra nodes to the analysis. The connectivity pattern does not change when more nodes are added to the analysis, which indicates the feasibility to analyze a high number of nodes using Granger-causality. This makes large-scale Granger-causality studies feasible and obviates the need to preselect nodes when analyzing high-density intracranial EEG, scalp EEG or MEG time series. This is important given the current clinical trend to use high density EEG (Michel and Murray 2012), MEG or stereo EEG (Alomar et al. 2016) to estimate the functional connectivity pattern.

Despite the fact that the developed methodology can localize the SOZ from a high number of intracranial EEG recordings, we must keep in mind that iEEG has a limited spatial sampling. If the SOZ is not sampled, the developed algorithm will not be able to localize it. This means that we can only estimate the connectivity pattern between the regions that are sampled.

In 2007, Ding et al. suggested to investigate the connectivity pattern in EEG source space, namely between the sources estimated from scalp EEG recordings (Ding et al. 2007). First the EEG signals are projected into source space by using EEG source imaging techniques and afterwards the connectivity pattern between the source signals is computed. Because the method investigates non-zero lag interactions in source space using a multivariate autoregressive model, it can cope with the problem of source leakage (Brookes et al. 2012). This technique has been used to extract whole brain connectivity patterns during interictal spikes, interictal resting state periods and ictal epoch from high-density scalp EEG measurements (Coito et al. 2015, 2016a; Staljanssens et al. 2017a) and to localize the seizure onset from ictal epoch recorded with standard clinical EEG setup (< 32 electrodes) (Staljanssens et al. 2017b). Until now the brain has been parcellated into a limited number of rather large brain regions to estimate the whole brain connectivity pattern. Our method enables to estimate whole brain connectivity pattern between many brain regions, allowing more detailed parcellation of the brain.

In future work we will estimate the SOZ in a large cohort of post-operatively seizure free and non-seizure-free patients to quantify the performance of the algorithm. Analyzing several seizures per patient would also allow us to test the reproducibility of our technique or the variability/extent of the SOZ, and its potential relationship with the seizure outcome. This is necessary before the method can be used in clinical practice.

## Conclusion

Our study showed that normalizing the time-series is an important pre-processing step that should not be overlooked, while adding more nodes to the analysis did only marginally affect the SOZ localization. This opens the way for high dimensional multivariate Granger-based connectivity analysis. For SOZ localization from intracranial EEG, ADTF analysis is more suitable than APDC analysis.

## Electronic supplementary material

Below is the link to the electronic supplementary material.

Supplementary material 1 (PDF 3742 KB)
