# Supplementary material for: Influence of Time-Series Normalization, Number of Nodes, Connectivity and Graph Measure Selection on Seizure-Onset Zone Localization from Intracranial EEG
Source: Brain Topogr. 2018 Apr 26;31(5):753–66. doi: 10.1007/s10548-018-0646-7 (PMC6097740; doi:10.1007/s10548-018-0646-7)
Supplement: Supplementary file 1 — Supplementary material 1 (PDF 3742 KB) [file 10548_2018_646_MOESM1_ESM.pdf]

Supplementary material

In the supplementary material the positions of the electrodes on the brain, the resection that rendered the patient seizure-free, the intracranial EEG during the seizure and the spectrograms of multiple channels are shown. The color coding of the channels corresponds to the color of the electrodes in the first figure.

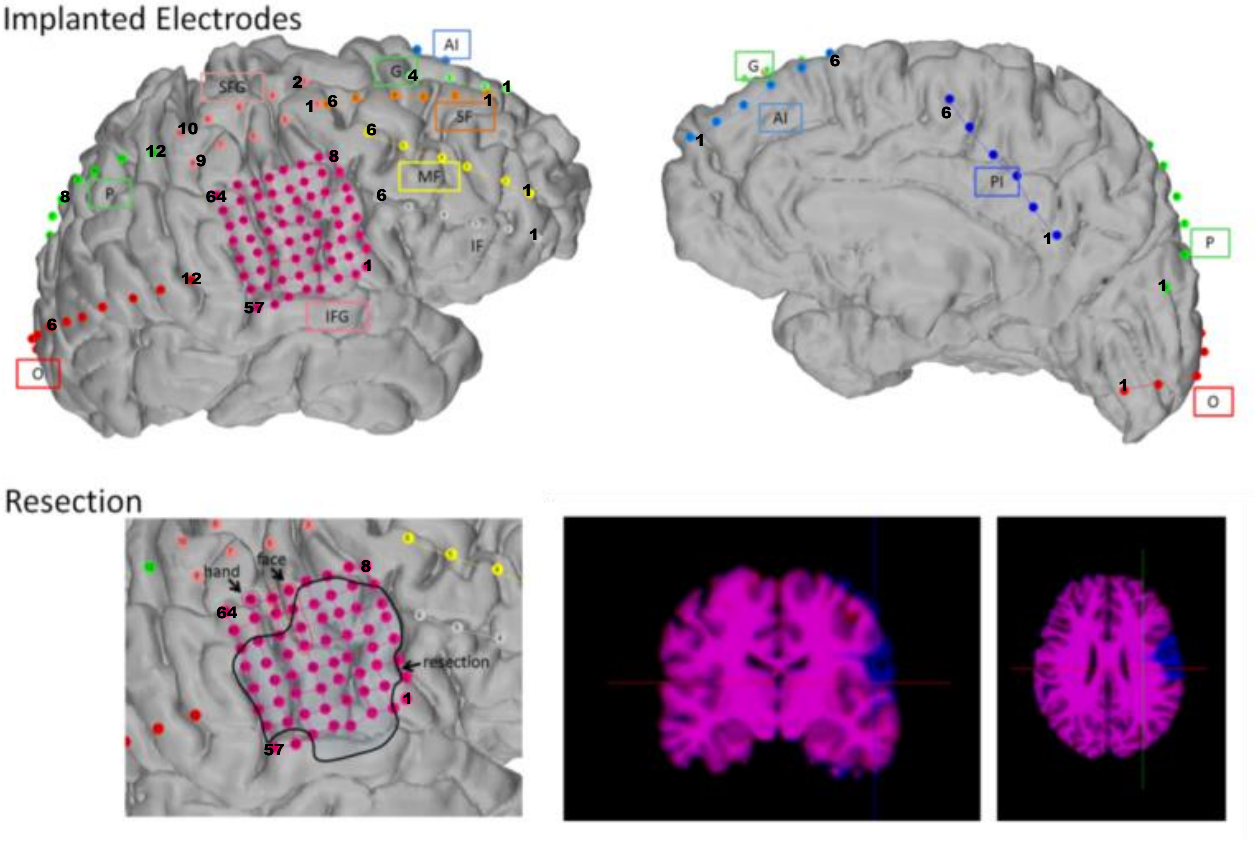

Intracranial EEG of the analyzed seizure

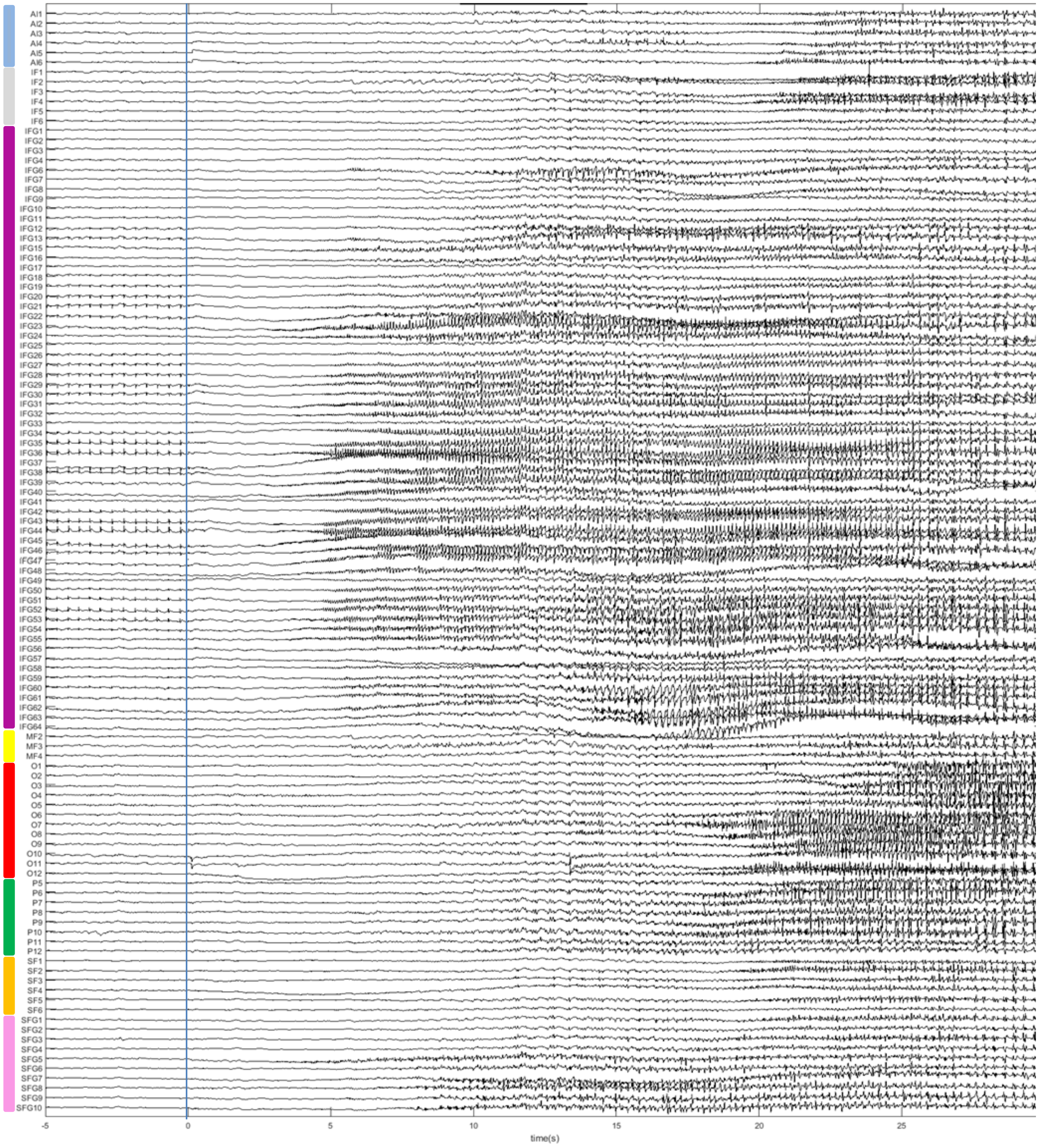

Spectrogram of a selection of channels during the seizure

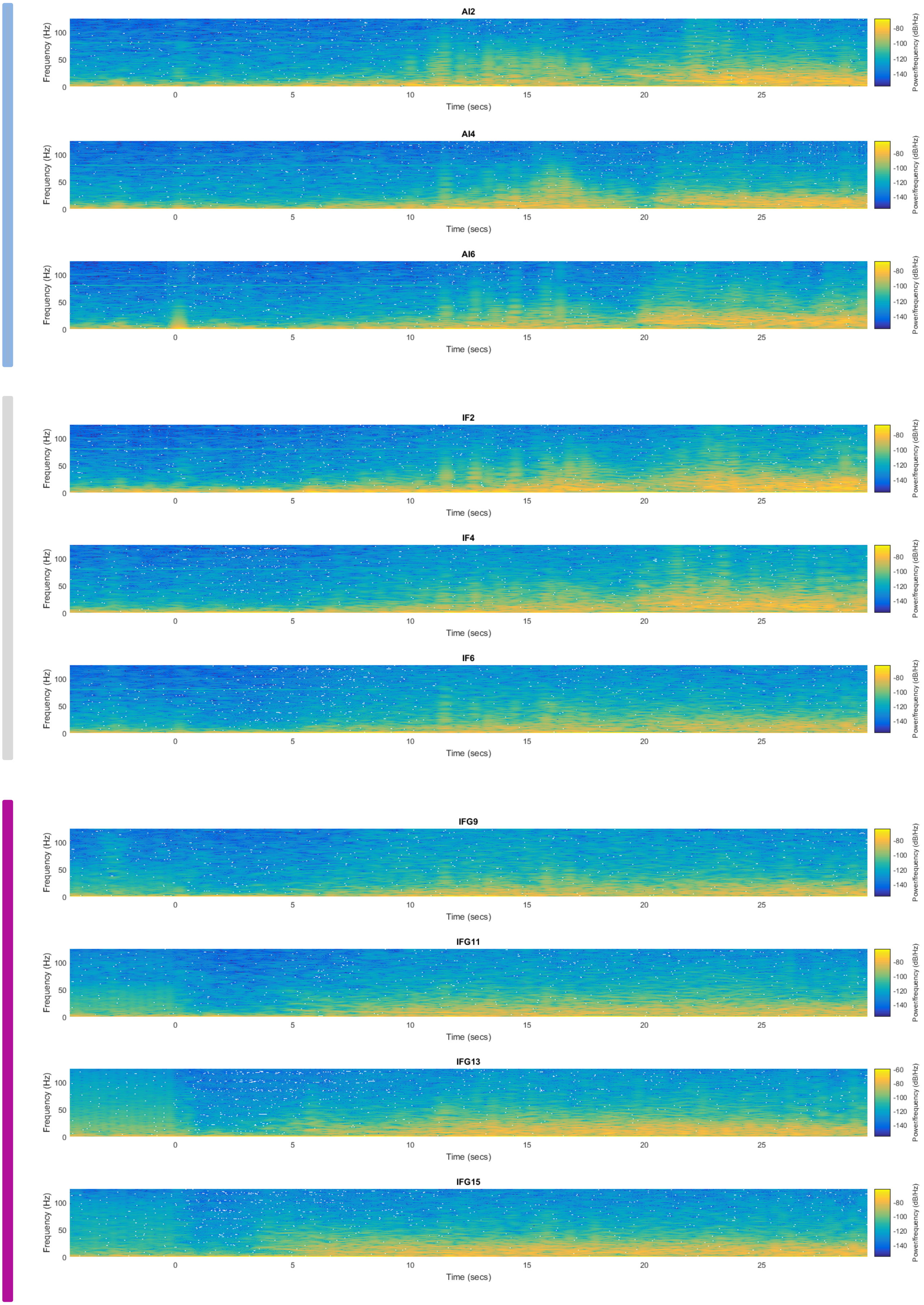

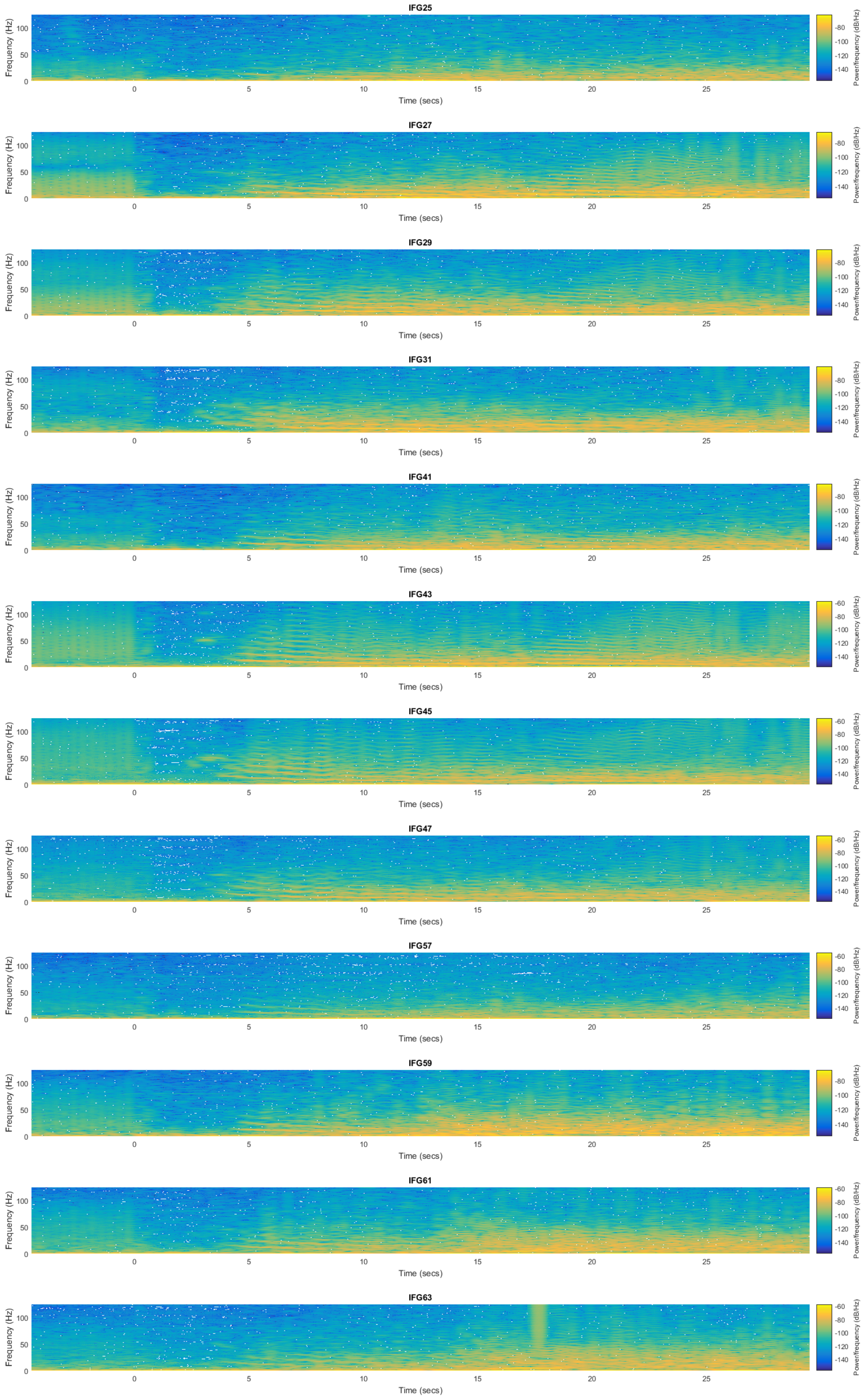
IFG25
IFG27
IFG29
IFG31
IFG41
IFG43
IFG45
IFG47
IFG57
IFG59
IFG61
IFG63
Frequency (Hz)
Time (secs)
Power/frequency (dB/Hz)

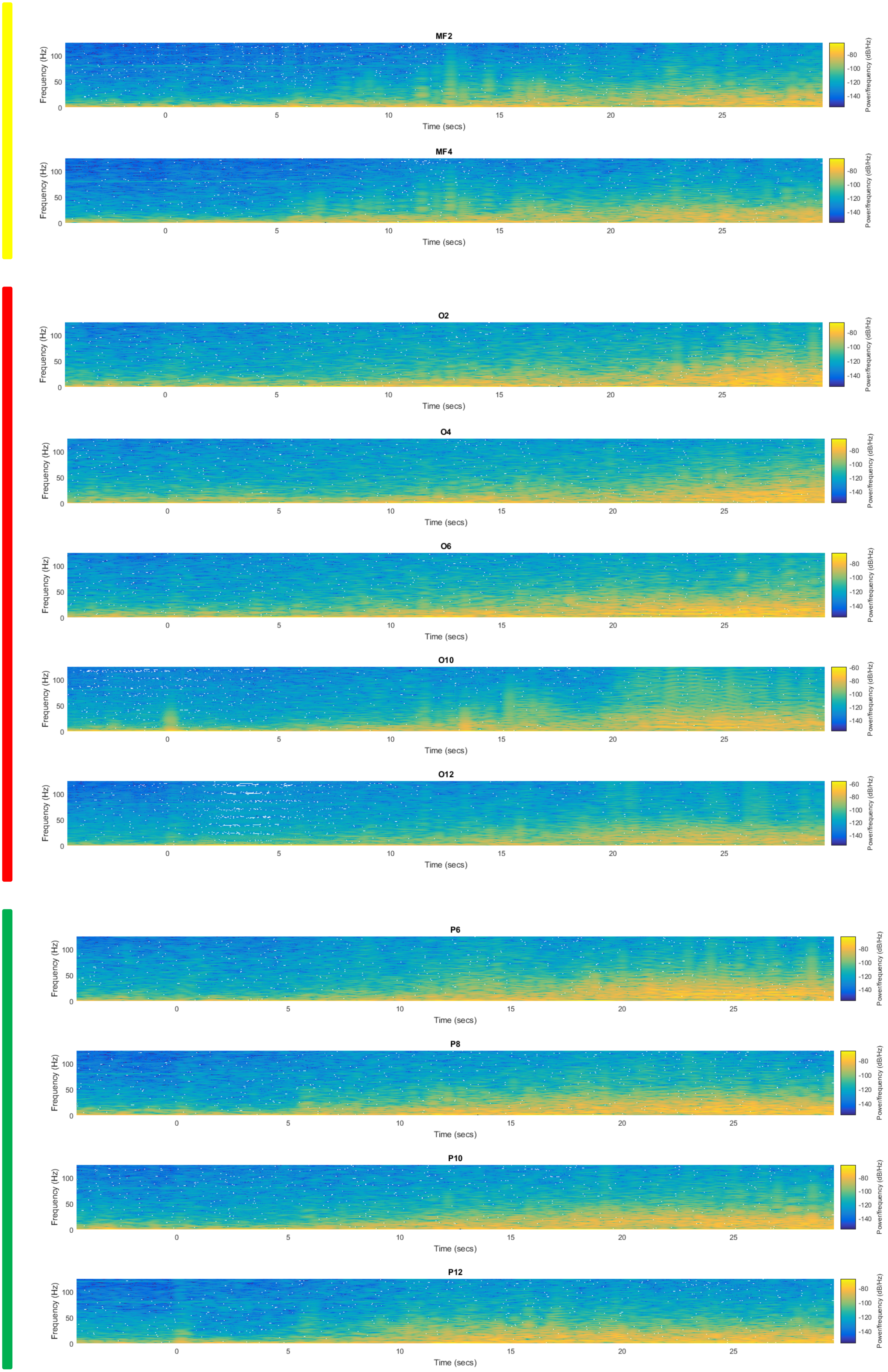
MF2
MF4
O2
O4
O6
O10
O12
P6
P8
P10
P12
Frequency (Hz)
Time (secs)
Power/frequency (dB/Hz)

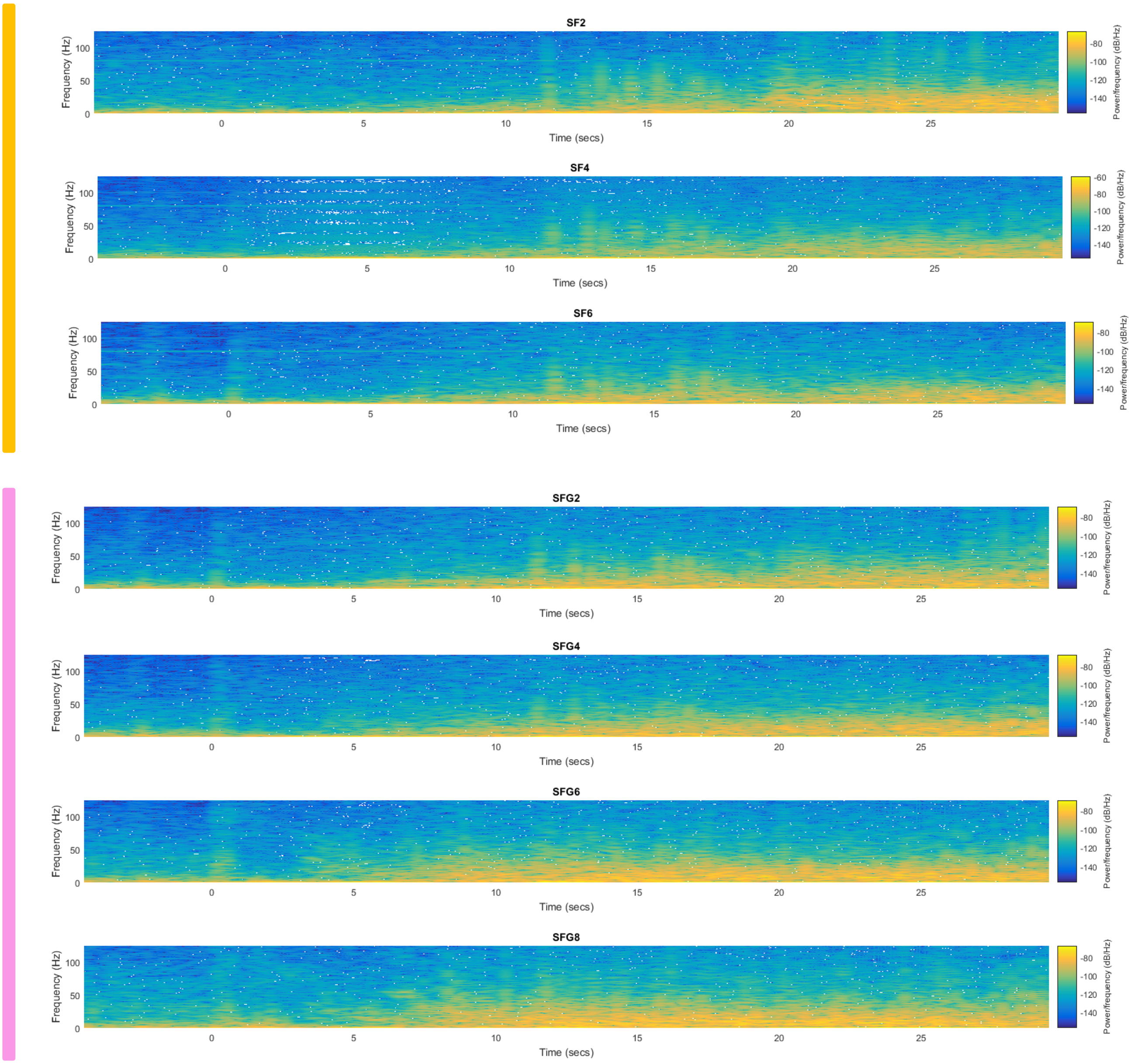
SF2
SF4
SF6
SFG2
SFG4
SFG6
SFG8
Frequency (Hz)
Time (secs)
Power/frequency (dB/Hz)
